# 5-Acetyl-4-(3-hy­droxy­phen­yl)-6-methyl-1,2,3,4-tetra­hydro­pyrimidin-2-one–tris­(hy­droxy­meth­yl)ammonium chloride (2/1)

**DOI:** 10.1107/S1600536813030559

**Published:** 2013-11-13

**Authors:** C. A. M. A. Huq, S. Fouzia, M. NizamMohideen

**Affiliations:** a PG & Research Department of Chemistry, The New College (Autonomous), Chennai 600 014, Tamilnadu, India; b Department of Physics, The New College (Autonomous), Chennai 600 014, Tamilnadu, India

## Abstract

The asymmetric unit of the title compound, 2C_13_H_14_N_2_O_3_·C_3_H_10_NO_3_^+^·Cl^−^, contains two independent mol­ecules (*A* and *B*) of the title pyrimidine derivative and one ion-pair of tris­(hy­droxy­meth­yl)ammonium chloride. The pyrimidine ring in each pyrimidine derivative has a half-chair conformation. Its mean plane is inclined to the benzene ring by 87.2 (3)° in mol­ecule *A* and 85.7 (2)° in mol­ecule *B*. In the crystal, the pyrimidine derivatives are connected to each other by N—H⋯O hydrogen bonds, forming chains propagating along the *b*-axis direction. The chains are linked *via* O—H—Cl hydrogen bonds, forming corrugated sheets lying parallel to the *bc* plane. The sheets are linked *via* C—H⋯O hydrogen bonds, forming a three-dimensional framework. The tris­(hy­droxy­meth­yl)ammonium chloride mol­ecules are located in the cages of the framework. There are also further C—H⋯O hydrogen bonds and C—H⋯π inter­actions present in the three-dimensional framework structure. Both the cation and chloride anion of the tris­(hy­droxy­meth­yl)ammonium chloride ion pair are disordered over two positions, with a refined occupancy ratio of 0.418 (8):0.582 (8) for the cation and 0.71 (4):0.29 (4) for the anion.

## Related literature

For the crystal structures of related pyrimidine derivatives, see: NizamMohideen *et al.* (2008*a* ▶,*b* ▶). For standard bond lengths, see: Allen *et al.* (1987 ▶). For puckering parameters, see: Cremer & Pople (1975 ▶). For asymmetry parameters, see: Nardelli (1983 ▶). For graph-set analysis, see: Bernstein *et al.* (1995 ▶).
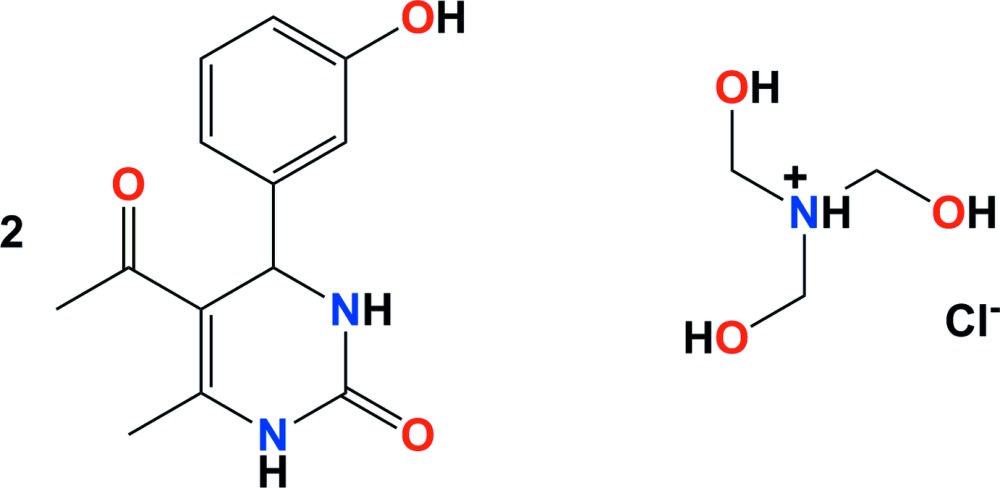


## Experimental

### 

#### Crystal data

2C_13_H_14_N_2_O_3_·C_3_H_10_NO_3_^+^·Cl^−^
*M* *_r_* = 636.09
Orthorhombic, 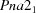

*a* = 15.7317 (7) Å
*b* = 7.2634 (12) Å
*c* = 28.8121 (3) Å
*V* = 3292.2 (6) Å^3^
*Z* = 4
Mo *K*α radiation
μ = 0.17 mm^−1^
*T* = 293 K
0.30 × 0.20 × 0.20 mm

#### Data collection

Bruker Kappa APEXII CCD diffractometer
Absorption correction: multi-scan (*SADABS*; Bruker, 2004 ▶) *T*_min_ = 0.950, *T*_max_ = 0.966
27037 measured reflections
5759 independent reflections
4284 reflections with *I* > 2σ(*I*)
*R*_int_ = 0.038

#### Refinement

*R*[*F* ^2^ > 2σ(*F* ^2^)] = 0.047
*wR*(*F* ^2^) = 0.157
*S* = 0.88
5759 reflections
489 parameters
270 restraints
H atoms treated by a mixture of independent and constrained refinement
Δρ_max_ = 0.21 e Å^−3^
Δρ_min_ = −0.23 e Å^−3^

### 

Data collection: *APEX2* (Bruker, 2004 ▶); cell refinement: *APEX2* and *SAINT* (Bruker, 2004 ▶); data reduction: *SAINT* and *XPREP* (Bruker, 2004 ▶); program(s) used to solve structure: *SHELXS97* (Sheldrick, 2008 ▶); program(s) used to refine structure: *SHELXL97* (Sheldrick, 2008 ▶); molecular graphics: *ORTEP-3 for Windows* (Farrugia, 2012 ▶) and *Mercury* (Macrae *et al.*, 2008 ▶); software used to prepare material for publication: *WinGX* (Farrugia, 2012 ▶) and *PLATON* (Spek, 2009 ▶).

## Supplementary Material

Crystal structure: contains datablock(s) global, I. DOI: 10.1107/S1600536813030559/su2662sup1.cif

Structure factors: contains datablock(s) I. DOI: 10.1107/S1600536813030559/su2662Isup2.hkl

Supplementary material file. DOI: 10.1107/S1600536813030559/su2662Isup3.cml

Additional supplementary materials:  crystallographic information; 3D view; checkCIF report

## Figures and Tables

**Table 1:** Hydrogen-bond geometry (Å, °) *Cg*1 and *Cg*2 are the centroids of the C8–C13 and C21–C26 rings, respectively.

*D*—H⋯*A*	*D*—H	H⋯*A*	*D*⋯*A*	*D*—H⋯*A*
N1—H1*A*⋯O4^i^	0.90 (3)	2.06 (4)	2.882 (6)	151 (4)
N2—H2*A*⋯O2^ii^	0.88 (2)	1.99 (3)	2.865 (5)	170 (5)
O3—H3⋯Cl1^iii^	0.82	2.26	3.044 (8)	161
N3—H3*A*⋯O5^ii^	0.87 (3)	1.97 (3)	2.839 (5)	175 (3)
N4—H4*A*⋯O1	0.88 (3)	2.03 (3)	2.836 (6)	152 (3)
O6—H6⋯Cl1	0.82	2.27	3.090 (7)	173
C13—H13⋯O1^i^	0.93	2.58	3.496 (7)	171
C18—H18*B*⋯O1^iv^	0.96	2.48	3.411 (7)	164
C22—H22⋯O4^i^	0.93	2.56	3.485 (6)	171
C28′—H28*D*⋯*Cg*1^v^	0.97	2.65	3.598 (17)	166
C27—H27*A*⋯*Cg*2^vi^	0.97	2.73	3.444 (19)	131

Symmetry codes: (i) 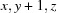; (ii) 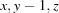; (iii) 
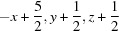
; (iv) 
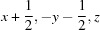
; (v) 
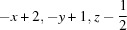
; (vi) 
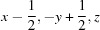
.

**Fig. 1.**
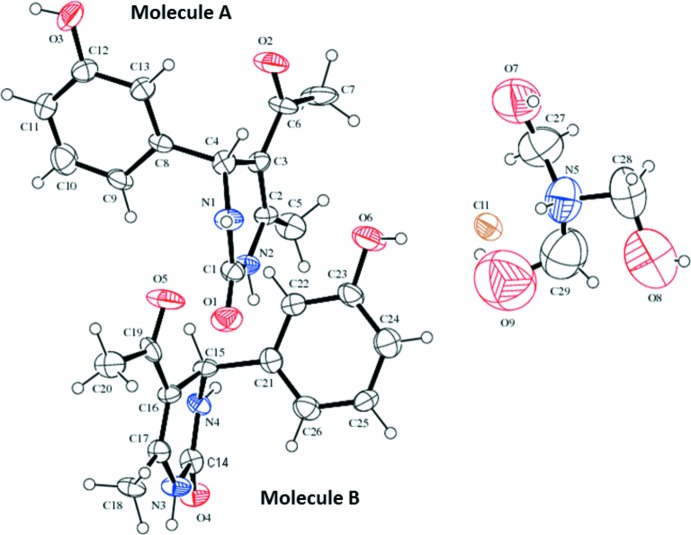
View of the molecular structure of the title compound, with atom labelling. Displacement ellipsoids are drawn at the 40% probability level. Only the major component of the tris(hydroxymethyl)ammonium chloride molecule is shown.

**Fig. 2.**
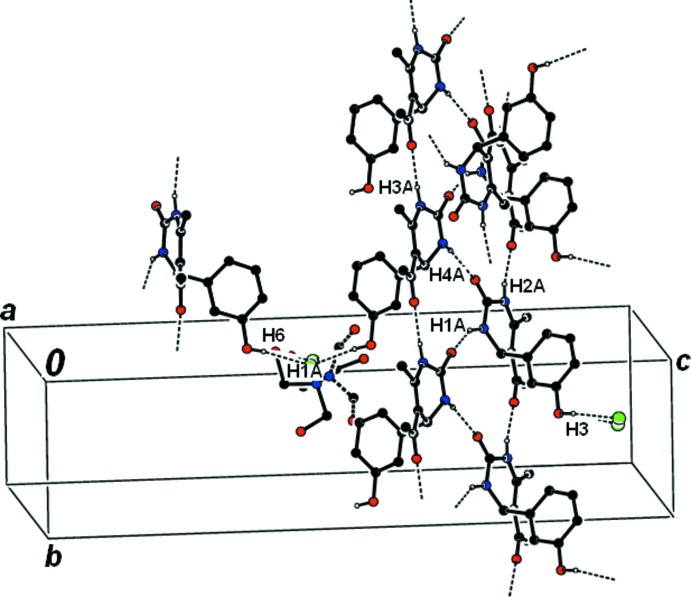
A view along the a axis of the crystal packing of the title compound. The hydrogen bonds are shown as dashed lines (see Table 1 for details; H atoms not involved in hydrogen bonding have been omitted for clarity).

## supplementary crystallographic information

### 1. Comment

As part of our ongoing investigations of pyrimidine derivatives (NizamMohideen *et al.*, 2008*a*,*b*), the title compound was synthesized and we report herein on its crystal structure.

In the asymmetric unit of the title compound there are two independent pyrimidine derivative molecules (A and B), and one nitrilotrimethanol hydrochloride molecule (Fig. 1). All bond lengths (Allen *et al.*, 1987) and angles in the pyrimidine derivative are within normal ranges and comparable with those in closely related structures (NizamMohideen *et al.*, 2008*a*,*b*). The normal probability plot analyses (International Tables for X-ray Crystallography, 1974, Vol. IV, pp. 293–309) for both bond lengths and angles show that the differences between the two symmetry independent molecules are of a statistical nature.

The dihydropyrimidine rings, (N1/N2/C1—C4) in molecule A and (N3/N4/C14—C17) in molecule B, adopt half-chair conformations. The puckering parameters (Cremer & Pople, 1975) and the lowest displacement asymmetry parameters (Nardelli, 1983), are q2 = 0.282 (5) Å, φ = 126.3 (10)°, θ = 108.4 (10)°, ΔS(C2/N1) is 16.9 (7)° and Δ2(N2/C4) is 15.8 (7)° for molecule A, and q2 =0.292 (5) Å, φ = 173.7 (9)°, θ = 72.0 (9)°, ΔS(C14/C16) is 18.4 (7)° and Δ2(N4/C17) is 16.1 (7)° for molecule B.

The dihedral angle between the mean plane of the pyrimidine ring and the benzene ring is 87.2 (3) in molecule A and 85.7 (2)° in molecule B. These values are close that of 86.5 (1)° found for Ethyl 6-methyl-2-oxo-4-phenyl-1,2,3,4-tetrahydropyrimidine-5-carboxylate (NizamMohideen *et al.*, 2008*a*).

The crystal packing is stabilized by strong N—H···O and O—H···Cl inter- and intramolecular hydrogen bonds and week intra- and intermolecular C—H···O and C—H···Cl interaction (Fig. 2 and Table 1). In both molecules, atoms O2 and O5 act donors in strong intermolecular N—H···O hydrogen bonds *via* H2A and H3A with the pyrimidine ring atoms N2 and N3, respectively, of a symmetry related molecule, generating a C(6) chain (Bernstein *et al.*, 1995). The interlinking of A and B molecules *via* strong N—H···O (N1—H1A···O4 and N4—H4A···O1) hydrogen bond generates infinite chains running along the *a* axis direction. The molecular packing is further stabilized by C—H···π interactions involving a methylene H atom of the tris(hydroxymethyl)ammonium cation and the pyrimidine ring of an adjacent molecule (Table 1). The crystal structure is further stabilized by O—H···Cl hydrogen bonds to form a three-dimensional supramolecular framework (Table 1 and Fig. 2).

The tris(hydroxymethyl)ammonium chloride molecule is disordered over two positions with refined occupancy ratios of 0.418 (8):0.528 (8) for the cation and 0.71 (4):0.29 (4) for the Cl^-^ anion. The geometry was regularized by soft restraints.

### 2. Experimental

A mixture of urea, 3-hydroxybenzaldehyde and acetyl acetone in the molar ratio 1.5:1:1 was ground in a mortar and pestle, in the presence of a catalytic amount of phosphoric acid, for 30 minutes, and then poured into a beaker containing ice cold water. The product obtained, 6-methyl-5-acetyl-3,4-dihydro-4-(3-hydroxyphenyl)-2(1*H*)-pyrimidinone [I], was filtered, washed with water and dried in air. Under ice cold conditions a few drops of triethylamine (0.01 mole) were added to chloroacetyl chloride (0.01 mole). 0.01 mol of [I] was added and then the mixture was stirred well for 10 min. Ethanol (20 ml) was then added and the mixture irradiated with microwave irradiation for 30 s. On evaporation of the solvent, the title solid product was obtained. It was recrystallized from ethanol giving brown block-like crystals suitable for X-Ray diffraction analysis.

### 3. Refinement

The tris(hydroxymethyl)ammonium chloride molecule is disordered both thermally and positionally; over two positions with refined occupancy ratios of 0.418 (8):0.528 (8) for the cation and 0.71 (4):0.29 (4) for the Cl^-^ anion. The corresponding bond distances involving the disordered atoms were restrained to be equal. The NH H atoms of the pyrimidine derivatives were located in difference Fourier maps and refined with *U*_iso_(H) = 1.2*U*_eq_(N). The OH, NH(cation) and C-bound H-atoms were placed in calculated positions and treated as riding atoms: O—H = 0.82 Å, N—H = 0.91 Å, C—H = 0.93–0.98 Å with *U*_iso_(H) = 1.5*U*_eq_(*C*-methyl and O) and = 1.2*U*_eq_(N,*C*) for other H atoms.

### Crystal data

**Table:**

2C_13_H_14_N_2_O_3_·C_3_H_10_NO_3_^+^·Cl^−^	*F*(000) = 1344
*M**_r_* = 636.09	*D*_x_ = 1.283 Mg m^−^^3^
Orthorhombic, *P**n**a*2_1_	Mo *K*α radiation, λ = 0.71073 Å
Hall symbol: P 2c -2n	Cell parameters from 5539 reflections
*a* = 15.7317 (7) Å	θ = 2.3–22.5°
*b* = 7.2634 (12) Å	µ = 0.17 mm^−^^1^
*c* = 28.8121 (3) Å	*T* = 293 K
*V* = 3292.2 (6) Å^3^	Block, brown
*Z* = 4	0.30 × 0.20 × 0.20 mm

### Data collection

**Table:**

Bruker Kappa APEXII CCD diffractometer	5759 independent reflections
Radiation source: fine-focus sealed tube	4284 reflections with *I* > 2σ(*I*)
Graphite monochromator	*R*_int_ = 0.038
ω and φ scans	θ_max_ = 25.0°, θ_min_ = 2.6°
Absorption correction: multi-scan (*SADABS*; Bruker, 2004)	*h* = −18→18
*T*_min_ = 0.950, *T*_max_ = 0.966	*k* = −8→8
27037 measured reflections	*l* = −34→33

### Refinement

**Table:**

Refinement on *F*^2^	Primary atom site location: structure-invariant direct methods
Least-squares matrix: full	Secondary atom site location: difference Fourier map
*R*[*F*^2^ > 2σ(*F*^2^)] = 0.047	Hydrogen site location: inferred from neighbouring sites
*wR*(*F*^2^) = 0.157	H atoms treated by a mixture of independent and constrained refinement
*S* = 0.88	*w* = 1/[σ^2^(*F*_o_^2^) + (0.1275*P*)^2^] where *P* = (*F*_o_^2^ + 2*F*_c_^2^)/3
5759 reflections	(Δ/σ)_max_ = 0.001
489 parameters	Δρ_max_ = 0.21 e Å^−^^3^
270 restraints	Δρ_min_ = −0.23 e Å^−^^3^

### Special details

**Table:**

Geometry. Bond distances, angles *etc*. have been calculated using the rounded fractional coordinates. All su's are estimated from the variances of the (full) variance-covariance matrix. The cell e.s.d.'s are taken into account in the estimation of distances, angles and torsion angles
Refinement. Refinement of *F*^2^ against ALL reflections. The weighted *R*-factor *wR* and goodness of fit *S* are based on *F*^2^, conventional *R*-factors *R* are based on *F*, with *F* set to zero for negative *F*^2^. The threshold expression of *F*^2^ > σ(*F*^2^) is used only for calculating *R*-factors(gt) *etc*. and is not relevant to the choice of reflections for refinement. *R*-factors based on *F*^2^ are statistically about twice as large as those based on *F*, and *R*-factors based on ALL data will be even larger.

### Fractional atomic coordinates and isotropic or equivalent isotropic displacement parameters (Å^2^)

**Table:**

	*x*	*y*	*z*	*U*_iso_*/*U*_eq_	Occ. (<1)
O1	1.1898 (3)	−0.1336 (5)	0.76654 (15)	0.0627 (17)	
O2	1.0190 (3)	0.5947 (4)	0.81444 (19)	0.0739 (16)	
O3	1.2755 (3)	0.7663 (6)	0.90241 (17)	0.0800 (19)	
N1	1.1703 (3)	0.1712 (6)	0.77776 (16)	0.0427 (14)	
N2	1.0738 (3)	−0.0308 (5)	0.80570 (14)	0.0394 (14)	
C1	1.1482 (3)	−0.0060 (5)	0.78268 (17)	0.0397 (16)	
C2	1.0148 (3)	0.1002 (5)	0.81631 (16)	0.0340 (16)	
C3	1.0405 (3)	0.2834 (6)	0.81304 (18)	0.0353 (16)	
C4	1.1346 (3)	0.3226 (6)	0.80297 (15)	0.0363 (14)	
C5	0.9322 (4)	0.0233 (7)	0.8310 (2)	0.0557 (19)	
C6	0.9843 (3)	0.4432 (6)	0.8171 (2)	0.0473 (18)	
C7	0.8944 (4)	0.4363 (7)	0.8239 (3)	0.080 (3)	
C8	1.1811 (3)	0.3670 (5)	0.84776 (16)	0.0330 (14)	
C9	1.2002 (4)	0.2298 (7)	0.8808 (2)	0.050 (2)	
C10	1.2412 (5)	0.2783 (10)	0.9205 (2)	0.067 (2)	
C11	1.2679 (4)	0.4511 (8)	0.92887 (18)	0.0463 (17)	
C12	1.2508 (4)	0.5852 (7)	0.89731 (19)	0.0483 (19)	
C13	1.2081 (3)	0.5437 (7)	0.8560 (2)	0.0410 (17)	
O4	1.3085 (2)	−0.6328 (4)	0.73448 (12)	0.0469 (11)	
O5	1.4787 (3)	0.0947 (4)	0.6874 (2)	0.0797 (19)	
O6	1.2233 (4)	0.2696 (6)	0.60050 (16)	0.082 (2)	
N3	1.4242 (3)	−0.5345 (5)	0.69651 (15)	0.0435 (14)	
N4	1.3262 (2)	−0.3263 (6)	0.72435 (15)	0.0380 (14)	
C14	1.3476 (3)	−0.4990 (6)	0.71941 (16)	0.0363 (16)	
C15	1.3671 (3)	−0.1749 (5)	0.69814 (15)	0.0373 (16)	
C16	1.4586 (3)	−0.2193 (5)	0.68982 (18)	0.0337 (16)	
C17	1.4817 (3)	−0.3934 (6)	0.68695 (18)	0.0380 (16)	
C18	1.5676 (3)	−0.4680 (6)	0.67216 (19)	0.0433 (16)	
C19	1.5110 (3)	−0.0543 (6)	0.6852 (2)	0.0417 (14)	
C20	1.6079 (3)	−0.0632 (7)	0.6794 (3)	0.063 (2)	
C21	1.3158 (3)	−0.1330 (7)	0.65405 (17)	0.0410 (17)	
C22	1.2883 (3)	0.0439 (7)	0.6458 (2)	0.0437 (17)	
C23	1.2456 (3)	0.0907 (7)	0.60659 (19)	0.0500 (19)	
C24	1.2280 (4)	−0.0445 (9)	0.5741 (2)	0.063 (2)	
C25	1.2538 (4)	−0.2274 (7)	0.5825 (2)	0.0483 (19)	
C26	1.2990 (4)	−0.2677 (8)	0.6224 (2)	0.0523 (19)	
O7'	1.0035 (13)	0.728 (2)	0.4720 (8)	0.292 (8)	0.582 (8)
O8'	0.9692 (11)	0.201 (2)	0.4311 (5)	0.196 (6)	0.582 (8)
O9'	0.9596 (13)	0.263 (3)	0.5715 (6)	0.229 (7)	0.582 (8)
N5'	0.9720 (5)	0.4167 (11)	0.4963 (4)	0.089 (3)	0.582 (8)
C27'	0.9651 (9)	0.6189 (16)	0.5113 (6)	0.164 (5)	0.582 (8)
C28'	0.9618 (11)	0.407 (2)	0.4438 (5)	0.155 (6)	0.582 (8)
C29'	0.9145 (11)	0.306 (2)	0.5242 (5)	0.162 (6)	0.582 (8)
O7	1.0080 (11)	0.673 (2)	0.5558 (6)	0.173 (6)	0.418 (8)
O8	0.9161 (16)	0.214 (3)	0.4526 (9)	0.243 (8)	0.418 (8)
O9	1.009 (2)	0.091 (3)	0.5610 (10)	0.293 (9)	0.418 (8)
N5	0.9777 (12)	0.3735 (18)	0.5171 (5)	0.123 (5)	0.418 (8)
C27	0.9570 (12)	0.509 (2)	0.5539 (7)	0.127 (6)	0.418 (8)
C28	0.9308 (18)	0.416 (3)	0.4721 (7)	0.155 (6)	0.418 (8)
C29	0.9450 (17)	0.178 (2)	0.5314 (8)	0.189 (7)	0.418 (8)
Cl1	1.1695 (4)	0.3682 (5)	0.5004 (2)	0.0599 (13)	0.71 (4)
Cl1'	1.1562 (16)	0.335 (4)	0.5030 (8)	0.105 (4)	0.29 (4)
H1A	1.2138 (19)	0.195 (6)	0.7584 (12)	0.0510*	
H2A	1.058 (3)	−0.147 (3)	0.8046 (17)	0.0470*	
H3	1.30050	0.77880	0.92720	0.0970*	
H4	1.13740	0.43170	0.78300	0.0440*	
H5A	0.88710	0.10050	0.81980	0.0840*	
H5B	0.93000	0.01770	0.86420	0.0840*	
H5C	0.92580	−0.09830	0.81840	0.0840*	
H7A	0.87180	0.55920	0.82390	0.1210*	
H7B	0.88220	0.37850	0.85310	0.1210*	
H7C	0.86870	0.36680	0.79930	0.1210*	
H9	1.18500	0.10790	0.87550	0.0600*	
H10	1.25110	0.18830	0.94280	0.0800*	
H11	1.29770	0.47850	0.95590	0.0560*	
H13	1.19790	0.63560	0.83430	0.0490*	
H3A	1.438 (3)	−0.650 (4)	0.6926 (18)	0.0520*	
H4A	1.2747 (15)	−0.298 (6)	0.7333 (12)	0.0460*	
H6	1.20900	0.28610	0.57340	0.0980*	
H15	1.36510	−0.06470	0.71780	0.0450*	
H18A	1.56130	−0.59270	0.66170	0.0650*	
H18B	1.60600	−0.46470	0.69810	0.0650*	
H18C	1.58990	−0.39370	0.64740	0.0650*	
H20A	1.63180	0.05660	0.68470	0.0940*	
H20B	1.62150	−0.10330	0.64860	0.0940*	
H20C	1.63110	−0.14870	0.70150	0.0940*	
H22	1.29940	0.13440	0.66780	0.0520*	
H24	1.19930	−0.01480	0.54690	0.0750*	
H25	1.24050	−0.32000	0.56140	0.0580*	
H26	1.31810	−0.38710	0.62770	0.0630*	
H5'	1.02570	0.37890	0.50300	0.1070*	0.582 (8)
H7'	1.05360	0.69870	0.46880	0.4380*	0.582 (8)
H8'	0.93090	0.14330	0.44390	0.2940*	0.582 (8)
H9'	0.96880	0.35990	0.58530	0.3440*	0.582 (8)
H27C	0.90620	0.65340	0.51610	0.1970*	0.582 (8)
H27D	0.99630	0.63980	0.53990	0.1970*	0.582 (8)
H28C	1.00590	0.47770	0.42850	0.1860*	0.582 (8)
H28D	0.90690	0.45520	0.43450	0.1860*	0.582 (8)
H29C	0.90130	0.19160	0.50820	0.1940*	0.582 (8)
H29D	0.86190	0.37180	0.52960	0.1940*	0.582 (8)
H5	1.03480	0.37080	0.51210	0.1480*	0.418 (8)
H7	1.02490	0.69820	0.52970	0.2600*	0.418 (8)
H8	0.89780	0.22040	0.42600	0.3640*	0.418 (8)
H9A	1.02430	0.16360	0.58100	0.4400*	0.418 (8)
H27A	0.89810	0.54520	0.55010	0.1530*	0.418 (8)
H27B	0.96140	0.44670	0.58370	0.1530*	0.418 (8)
H28A	0.96520	0.48940	0.45120	0.1870*	0.418 (8)
H28B	0.87730	0.47860	0.47790	0.1870*	0.418 (8)
H29A	0.89170	0.18930	0.54810	0.2270*	0.418 (8)
H29B	0.93540	0.10410	0.50390	0.2270*	0.418 (8)

### Atomic displacement parameters (Å^2^)

**Table:**

	*U*^11^	*U*^22^	*U*^33^	*U*^12^	*U*^13^	*U*^23^
O1	0.061 (3)	0.059 (3)	0.068 (3)	0.0137 (19)	0.012 (2)	−0.020 (2)
O2	0.053 (2)	0.0307 (19)	0.138 (4)	0.0040 (16)	−0.002 (3)	−0.003 (2)
O3	0.107 (4)	0.056 (3)	0.077 (3)	−0.022 (2)	−0.041 (3)	−0.006 (2)
N1	0.040 (2)	0.039 (2)	0.049 (3)	0.0013 (18)	0.0200 (19)	−0.0030 (19)
N2	0.042 (2)	0.0253 (19)	0.051 (3)	−0.0083 (16)	0.007 (2)	0.0034 (18)
C1	0.042 (3)	0.034 (2)	0.043 (3)	−0.001 (2)	0.002 (2)	0.006 (2)
C2	0.042 (3)	0.027 (2)	0.033 (3)	−0.0009 (18)	0.006 (2)	−0.0004 (19)
C3	0.031 (3)	0.033 (2)	0.042 (3)	−0.0068 (18)	0.003 (2)	−0.003 (2)
C4	0.025 (2)	0.040 (2)	0.044 (3)	−0.0119 (18)	0.008 (2)	0.007 (2)
C5	0.042 (3)	0.049 (3)	0.076 (4)	−0.006 (2)	0.012 (3)	0.008 (3)
C6	0.054 (3)	0.021 (2)	0.067 (4)	0.008 (2)	0.007 (3)	0.006 (2)
C7	0.053 (4)	0.040 (3)	0.148 (6)	0.014 (2)	0.004 (4)	−0.035 (3)
C8	0.026 (2)	0.026 (2)	0.047 (3)	0.0025 (16)	0.010 (2)	0.005 (2)
C9	0.056 (4)	0.028 (3)	0.067 (4)	0.006 (2)	−0.008 (3)	0.008 (3)
C10	0.075 (4)	0.077 (4)	0.048 (4)	0.008 (3)	−0.003 (3)	0.009 (3)
C11	0.047 (3)	0.054 (3)	0.038 (3)	−0.004 (2)	−0.004 (2)	−0.004 (3)
C12	0.055 (4)	0.045 (3)	0.045 (3)	0.002 (2)	0.004 (3)	−0.009 (2)
C13	0.040 (3)	0.038 (3)	0.045 (3)	−0.010 (2)	0.004 (2)	0.002 (2)
O4	0.042 (2)	0.0386 (19)	0.060 (2)	−0.0107 (14)	0.0150 (17)	−0.0007 (17)
O5	0.049 (2)	0.0261 (19)	0.164 (5)	0.0039 (16)	0.005 (3)	−0.005 (2)
O6	0.131 (5)	0.055 (3)	0.060 (3)	0.037 (3)	−0.003 (3)	0.002 (2)
N3	0.037 (2)	0.0276 (19)	0.066 (3)	−0.0065 (16)	0.015 (2)	0.002 (2)
N4	0.029 (2)	0.040 (2)	0.045 (3)	0.0097 (16)	0.0058 (17)	−0.0005 (19)
C14	0.028 (2)	0.045 (3)	0.036 (3)	−0.007 (2)	0.009 (2)	0.010 (2)
C15	0.043 (3)	0.021 (2)	0.048 (3)	−0.0082 (18)	0.002 (2)	0.0019 (19)
C16	0.031 (3)	0.026 (2)	0.044 (3)	−0.0033 (17)	0.004 (2)	−0.0038 (19)
C17	0.023 (2)	0.040 (3)	0.051 (3)	0.0007 (17)	0.003 (2)	−0.001 (2)
C18	0.037 (3)	0.030 (2)	0.063 (3)	0.0072 (19)	0.013 (3)	0.001 (2)
C19	0.024 (2)	0.042 (2)	0.059 (3)	0.0027 (19)	−0.007 (2)	0.015 (2)
C20	0.026 (3)	0.055 (3)	0.108 (5)	−0.003 (2)	0.006 (3)	−0.012 (3)
C21	0.029 (3)	0.050 (3)	0.044 (3)	0.002 (2)	0.005 (2)	0.001 (2)
C22	0.046 (3)	0.038 (3)	0.047 (3)	−0.001 (2)	0.002 (2)	−0.004 (2)
C23	0.044 (3)	0.051 (3)	0.055 (4)	0.019 (2)	0.003 (3)	−0.002 (2)
C24	0.057 (4)	0.068 (4)	0.063 (4)	0.002 (3)	0.000 (3)	0.003 (3)
C25	0.053 (3)	0.038 (3)	0.054 (4)	−0.004 (2)	−0.005 (3)	−0.012 (2)
C26	0.057 (4)	0.049 (3)	0.051 (3)	0.014 (2)	0.006 (3)	−0.001 (3)
O7'	0.300 (15)	0.273 (14)	0.303 (15)	0.041 (11)	0.023 (11)	0.032 (11)
O8'	0.179 (10)	0.264 (11)	0.144 (9)	0.084 (9)	−0.033 (8)	−0.031 (8)
O9'	0.216 (12)	0.293 (13)	0.179 (10)	0.038 (11)	0.014 (9)	0.013 (10)
N5'	0.052 (3)	0.122 (5)	0.094 (7)	0.026 (4)	−0.013 (5)	0.019 (6)
C27'	0.145 (8)	0.169 (9)	0.177 (11)	0.073 (7)	−0.024 (8)	−0.055 (8)
C28'	0.123 (9)	0.208 (11)	0.135 (10)	0.047 (9)	−0.058 (8)	0.033 (9)
C29'	0.149 (10)	0.202 (12)	0.134 (8)	−0.014 (9)	0.049 (7)	−0.004 (9)
O7	0.172 (11)	0.180 (10)	0.168 (11)	0.049 (9)	0.016 (9)	−0.031 (9)
O8	0.220 (14)	0.271 (14)	0.237 (15)	0.031 (12)	−0.010 (12)	0.036 (12)
O9	0.290 (16)	0.301 (16)	0.289 (16)	0.052 (13)	0.012 (13)	−0.013 (13)
N5	0.117 (8)	0.156 (8)	0.097 (8)	−0.018 (7)	−0.001 (7)	0.011 (7)
C27	0.106 (9)	0.138 (10)	0.138 (10)	0.006 (8)	0.033 (8)	−0.028 (8)
C28	0.123 (11)	0.207 (11)	0.136 (11)	0.022 (10)	−0.025 (10)	0.021 (10)
C29	0.190 (12)	0.213 (12)	0.164 (11)	−0.001 (10)	−0.001 (10)	−0.021 (10)
Cl1	0.075 (2)	0.063 (3)	0.0417 (17)	0.0184 (10)	0.0047 (18)	0.0032 (18)
Cl1'	0.062 (4)	0.174 (9)	0.080 (5)	0.046 (6)	0.023 (6)	0.036 (7)

### Geometric parameters (Å, º)

**Table:**

O1—C1	1.226 (6)	C10—C11	1.345 (9)
O2—C6	1.231 (6)	C11—C12	1.359 (8)
O3—C12	1.379 (7)	C12—C13	1.400 (8)
O3—H3	0.8200	C4—H4	0.9800
O4—C14	1.229 (5)	C5—H5C	0.9600
O5—C19	1.197 (6)	C5—H5A	0.9600
O6—C23	1.357 (7)	C5—H5B	0.9600
O6—H6	0.8200	C7—H7B	0.9600
O7'—C27'	1.51 (3)	C7—H7C	0.9600
O8'—C28'	1.55 (2)	C7—H7A	0.9600
O9'—C29'	1.57 (2)	C9—H9	0.9300
O7'—H7'	0.8200	C10—H10	0.9300
O8'—H8'	0.8200	C11—H11	0.9300
O9'—H9'	0.8200	C13—H13	0.9300
O7—C27	1.44 (2)	C15—C16	1.495 (7)
O8—C28	1.59 (3)	C15—C21	1.536 (7)
O9—C29	1.46 (4)	C16—C17	1.318 (6)
O7—H7	0.8200	C16—C19	1.461 (6)
O8—H8	0.8200	C17—C18	1.517 (7)
O9—H9A	0.8200	C19—C20	1.535 (7)
N1—C4	1.433 (6)	C21—C26	1.363 (8)
N1—C1	1.341 (6)	C21—C22	1.377 (7)
N2—C1	1.357 (7)	C22—C23	1.358 (8)
N2—C2	1.364 (6)	C23—C24	1.385 (8)
N1—H1A	0.90 (3)	C24—C25	1.410 (8)
N2—H2A	0.88 (2)	C25—C26	1.383 (8)
N3—C14	1.398 (7)	C15—H15	0.9800
N3—C17	1.394 (6)	C18—H18C	0.9600
N4—C15	1.481 (6)	C18—H18A	0.9600
N4—C14	1.307 (6)	C18—H18B	0.9600
N3—H3A	0.87 (3)	C20—H20B	0.9600
N4—H4A	0.88 (3)	C20—H20A	0.9600
N5'—C27'	1.535 (15)	C20—H20C	0.9600
N5'—C29'	1.453 (18)	C22—H22	0.9300
N5'—C28'	1.523 (18)	C24—H24	0.9300
N5'—H5'	0.9100	C25—H25	0.9300
N5—C28	1.52 (3)	C26—H26	0.9300
N5—C27	1.48 (2)	C27'—H27C	0.9700
N5—C29	1.57 (2)	C27'—H27D	0.9700
N5—H5	0.9100	C28'—H28C	0.9700
C2—C5	1.476 (8)	C28'—H28D	0.9700
C2—C3	1.394 (6)	C29'—H29C	0.9700
C3—C6	1.464 (6)	C29'—H29D	0.9700
C3—C4	1.535 (7)	C27—H27A	0.9700
C4—C8	1.518 (6)	C27—H27B	0.9700
C6—C7	1.429 (8)	C28—H28A	0.9700
C8—C9	1.411 (7)	C28—H28B	0.9700
C8—C13	1.373 (6)	C29—H29A	0.9700
C9—C10	1.360 (9)	C29—H29B	0.9700
			
C12—O3—H3	109.00	N3—C14—N4	116.8 (4)
C23—O6—H6	109.00	C16—C15—C21	114.6 (4)
C27'—O7'—H7'	109.00	N4—C15—C21	109.9 (4)
C28'—O8'—H8'	109.00	N4—C15—C16	109.9 (3)
C29'—O9'—H9'	109.00	C17—C16—C19	128.7 (4)
C27—O7—H7	109.00	C15—C16—C17	118.9 (4)
C28—O8—H8	109.00	C15—C16—C19	112.4 (3)
C29—O9—H9A	110.00	C16—C17—C18	127.3 (4)
C1—N1—C4	125.6 (4)	N3—C17—C18	111.8 (4)
C1—N2—C2	127.1 (4)	N3—C17—C16	120.9 (4)
C1—N1—H1A	117 (3)	O5—C19—C16	119.8 (5)
C4—N1—H1A	118 (3)	O5—C19—C20	117.7 (4)
C1—N2—H2A	111 (3)	C16—C19—C20	122.4 (4)
C2—N2—H2A	119 (3)	C15—C21—C22	119.5 (4)
C14—N3—C17	121.1 (4)	C22—C21—C26	119.6 (5)
C14—N4—C15	123.1 (4)	C15—C21—C26	120.9 (4)
C14—N3—H3A	117 (3)	C21—C22—C23	122.2 (5)
C17—N3—H3A	121 (3)	C22—C23—C24	118.9 (5)
C15—N4—H4A	112 (3)	O6—C23—C24	122.7 (5)
C14—N4—H4A	120 (3)	O6—C23—C22	118.4 (5)
C28'—N5'—C29'	117.3 (10)	C23—C24—C25	119.6 (5)
C27'—N5'—C29'	109.3 (10)	C24—C25—C26	119.3 (5)
C27'—N5'—C28'	108.5 (10)	C21—C26—C25	120.3 (5)
C27'—N5'—H5'	107.00	C16—C15—H15	107.00
C29'—N5'—H5'	107.00	C21—C15—H15	107.00
C28'—N5'—H5'	107.00	N4—C15—H15	107.00
C27—N5—C28	111.6 (15)	H18A—C18—H18C	110.00
C27—N5—C29	110.0 (14)	C17—C18—H18C	109.00
C28—N5—C29	104.4 (16)	H18A—C18—H18B	109.00
C29—N5—H5	110.00	C17—C18—H18B	109.00
C28—N5—H5	110.00	C17—C18—H18A	109.00
C27—N5—H5	110.00	H18B—C18—H18C	109.00
O1—C1—N2	123.0 (4)	H20B—C20—H20C	109.00
O1—C1—N1	123.2 (5)	C19—C20—H20B	110.00
N1—C1—N2	113.8 (4)	H20A—C20—H20C	109.00
N2—C2—C5	113.5 (4)	C19—C20—H20C	109.00
C3—C2—C5	129.5 (4)	C19—C20—H20A	109.00
N2—C2—C3	117.0 (4)	H20A—C20—H20B	110.00
C2—C3—C6	125.2 (4)	C23—C22—H22	119.00
C4—C3—C6	116.8 (4)	C21—C22—H22	119.00
C2—C3—C4	118.0 (4)	C25—C24—H24	120.00
N1—C4—C3	109.4 (4)	C23—C24—H24	120.00
N1—C4—C8	113.9 (4)	C26—C25—H25	120.00
C3—C4—C8	110.1 (4)	C24—C25—H25	120.00
C3—C6—C7	125.5 (4)	C21—C26—H26	120.00
O2—C6—C3	115.9 (4)	C25—C26—H26	120.00
O2—C6—C7	118.6 (4)	O7'—C27'—N5'	105.3 (12)
C4—C8—C13	119.7 (4)	O8'—C28'—N5'	105.8 (11)
C9—C8—C13	118.6 (5)	O9'—C29'—N5'	108.0 (13)
C4—C8—C9	121.8 (4)	H27C—C27'—H27D	109.00
C8—C9—C10	119.1 (5)	N5'—C27'—H27D	111.00
C9—C10—C11	122.7 (6)	N5'—C27'—H27C	111.00
C10—C11—C12	119.1 (5)	O7'—C27'—H27C	111.00
O3—C12—C11	123.8 (5)	O7'—C27'—H27D	111.00
C11—C12—C13	120.6 (5)	O8'—C28'—H28C	111.00
O3—C12—C13	115.5 (5)	H28C—C28'—H28D	109.00
C8—C13—C12	119.8 (5)	N5'—C28'—H28C	111.00
N1—C4—H4	108.00	O8'—C28'—H28D	111.00
C8—C4—H4	108.00	N5'—C28'—H28D	111.00
C3—C4—H4	108.00	N5'—C29'—H29C	110.00
C2—C5—H5C	109.00	N5'—C29'—H29D	110.00
H5A—C5—H5C	109.00	O9'—C29'—H29C	110.00
C2—C5—H5B	110.00	O9'—C29'—H29D	110.00
C2—C5—H5A	109.00	H29C—C29'—H29D	108.00
H5A—C5—H5B	109.00	O7—C27—N5	117.1 (16)
H5B—C5—H5C	110.00	O8—C28—N5	100.6 (16)
H7A—C7—H7B	109.00	O9—C29—N5	108.6 (19)
C6—C7—H7B	109.00	O7—C27—H27A	108.00
H7B—C7—H7C	109.00	O7—C27—H27B	108.00
C6—C7—H7C	110.00	N5—C27—H27A	108.00
C6—C7—H7A	109.00	N5—C27—H27B	108.00
H7A—C7—H7C	109.00	H27A—C27—H27B	107.00
C8—C9—H9	120.00	O8—C28—H28A	112.00
C10—C9—H9	120.00	O8—C28—H28B	112.00
C9—C10—H10	119.00	N5—C28—H28A	112.00
C11—C10—H10	119.00	N5—C28—H28B	112.00
C10—C11—H11	120.00	H28A—C28—H28B	109.00
C12—C11—H11	120.00	O9—C29—H29A	110.00
C12—C13—H13	120.00	O9—C29—H29B	110.00
C8—C13—H13	120.00	N5—C29—H29A	110.00
O4—C14—N3	116.9 (4)	N5—C29—H29B	110.00
O4—C14—N4	126.3 (4)	H29A—C29—H29B	109.00
			
C4—N1—C1—O1	−167.0 (5)	C3—C4—C8—C13	−109.3 (5)
C4—N1—C1—N2	15.3 (7)	C4—C8—C9—C10	−179.3 (5)
C1—N1—C4—C3	−33.4 (6)	C9—C8—C13—C12	−2.3 (8)
C1—N1—C4—C8	90.3 (6)	C4—C8—C13—C12	179.8 (5)
C2—N2—C1—O1	−165.3 (5)	C13—C8—C9—C10	2.9 (8)
C1—N2—C2—C5	165.7 (5)	C8—C9—C10—C11	−3.0 (10)
C1—N2—C2—C3	−15.8 (7)	C9—C10—C11—C12	2.5 (11)
C2—N2—C1—N1	12.4 (7)	C10—C11—C12—C13	−1.8 (9)
C14—N3—C17—C16	−14.9 (7)	C10—C11—C12—O3	−179.8 (6)
C17—N3—C14—N4	11.1 (7)	C11—C12—C13—C8	1.8 (8)
C17—N3—C14—O4	−166.2 (4)	O3—C12—C13—C8	179.9 (5)
C14—N3—C17—C18	167.0 (4)	N4—C15—C21—C22	125.6 (5)
C14—N4—C15—C21	93.6 (5)	N4—C15—C21—C26	−56.0 (6)
C14—N4—C15—C16	−33.4 (6)	C21—C15—C16—C19	83.2 (5)
C15—N4—C14—O4	−168.3 (4)	C21—C15—C16—C17	−95.9 (5)
C15—N4—C14—N3	14.6 (6)	N4—C15—C16—C19	−152.5 (4)
C27'—N5'—C29'—O9'	−82.1 (14)	C16—C15—C21—C22	−110.1 (5)
C27'—N5'—C28'—O8'	−179.9 (11)	C16—C15—C21—C26	68.3 (6)
C29'—N5'—C27'—O7'	−162.5 (13)	N4—C15—C16—C17	28.4 (6)
C28'—N5'—C29'—O9'	154.1 (12)	C15—C16—C19—O5	−1.3 (8)
C29'—N5'—C28'—O8'	−55.6 (16)	C15—C16—C17—C18	170.8 (5)
C28'—N5'—C27'—O7'	−33.6 (15)	C15—C16—C17—N3	−7.0 (8)
C5—C2—C3—C6	−10.1 (9)	C17—C16—C19—O5	177.7 (6)
C5—C2—C3—C4	171.8 (5)	C17—C16—C19—C20	−4.8 (9)
N2—C2—C3—C6	171.7 (5)	C15—C16—C19—C20	176.3 (5)
N2—C2—C3—C4	−6.4 (7)	C19—C16—C17—C18	−8.2 (9)
C2—C3—C6—C7	−1.6 (9)	C19—C16—C17—N3	174.1 (5)
C2—C3—C6—O2	178.2 (5)	C15—C21—C26—C25	−179.0 (5)
C4—C3—C6—C7	176.5 (6)	C26—C21—C22—C23	−1.0 (8)
C4—C3—C6—O2	−3.7 (7)	C15—C21—C22—C23	177.4 (5)
C6—C3—C4—N1	−151.0 (4)	C22—C21—C26—C25	−0.7 (8)
C6—C3—C4—C8	83.2 (5)	C21—C22—C23—C24	1.0 (8)
C2—C3—C4—N1	27.3 (6)	C21—C22—C23—O6	−177.8 (5)
C2—C3—C4—C8	−98.6 (5)	O6—C23—C24—C25	179.4 (6)
N1—C4—C8—C9	−50.3 (6)	C22—C23—C24—C25	0.7 (8)
N1—C4—C8—C13	127.5 (5)	C23—C24—C25—C26	−2.3 (9)
C3—C4—C8—C9	73.0 (6)	C24—C25—C26—C21	2.3 (9)

### Hydrogen-bond geometry (Å, º)

Cg1 and Cg2 are the centroids of the C8–C13 and C21–C26 rings, respectively.

**Table:**

*D*—H···*A*	*D*—H	H···*A*	*D*···*A*	*D*—H···*A*
N1—H1*A*···O4^i^	0.90 (3)	2.06 (4)	2.882 (6)	151 (4)
N2—H2*A*···O2^ii^	0.88 (2)	1.99 (3)	2.865 (5)	170 (5)
O3—H3···Cl1^iii^	0.82	2.26	3.044 (8)	161
N3—H3*A*···O5^ii^	0.87 (3)	1.97 (3)	2.839 (5)	175 (3)
N4—H4*A*···O1	0.88 (3)	2.03 (3)	2.836 (6)	152 (3)
O6—H6···Cl1	0.82	2.27	3.090 (7)	173
C13—H13···O1^i^	0.93	2.58	3.496 (7)	171
C18—H18*B*···O1^iv^	0.96	2.48	3.411 (7)	164
C22—H22···O4^i^	0.93	2.56	3.485 (6)	171
C28′—H28*D*···*Cg*1^v^	0.97	2.65	3.598 (17)	166
C27—H27*A*···*Cg*2^vi^	0.97	2.73	3.444 (19)	131

Symmetry codes: (i) *x*, *y*+1, *z*; (ii) *x*, *y*−1, *z*; (iii) −*x*+5/2, *y*+1/2, *z*+1/2; (iv) *x*+1/2, −*y*−1/2, *z*; (v) −*x*+2, −*y*+1, *z*−1/2; (vi) *x*−1/2, −*y*+1/2, *z*.
